# The “Direct tip injection in occlusive lesions (DIOL)” fashion

**DOI:** 10.1186/s42155-021-00276-w

**Published:** 2021-12-14

**Authors:** Takuya Haraguchi, Tsutomu Fujita, Yoshifumi Kashima, Masanaga Tsujimoto, Tomohiko Watanabe, Takuro Sugie, Daisuke Hachinohe, Umihiko Kaneko, Ken Kobayashi, Daitaro Kanno, Katsuhiko Sato

**Affiliations:** Director of Cardiology and Head of Peripheral Artery Disease Center, Sapporo Heart Center, North 49, East 16, 8-1, Higashi ward, Sapporo, Hokkaido 007-0849 Japan

**Keywords:** Chronic total occlusions, Calcification, Endovascular intervention, Peripheral arterial disease, Femoropopliteal artery disease, Below-the-knee artery disease, Critical limb-threatening ischemia, Intermittent claudication

## Abstract

**Background:**

The successful intervention for peripheral artery disease is limited by complex chronic total occlusions (CTOs). During CTO wiring, without the use of intravascular or extravascular ultrasound, the guidewire position is unclear, except for calcified lesions showing the vessel path. To solve this problem, we propose a novel guidewire crossing with plaque modification method for complex occlusive lesions, named the “Direct tip Injection in Occlusive Lesions (DIOL)” fashion.

**Main text:**

The “DIOL” fashion utilizes the hydraulic pressure of tip injection with a general contrast media through a microcatheter or an over-the-wire balloon catheter within CTOs. The purposes of this technique are 1) to visualize the “vessel road” of the occlusion from expanding a microchannel, subintimal, intramedial, and periadventitial space with contrast agent and 2) to modify plaques within CTO to advance CTO devices safely and easily. This technique creates dissections by hydraulic pressure. Antegrade-DIOL may create dissections which extend to and compress a distal lumen, especially in below-the-knee arteries. A gentle tip injection with smaller contrast volume (1–2 ml) should be used to confirm the tip position which is inside or outside of a vessel. On the other hand, retrograde-DIOL is used with a forceful tip injection of moderate contrast volume up to 5-ml to visualize vessel tracks and to modify the plaques to facilitate the crossing of CTO devices. Case-1 involved a severe claudicant due to right superficial femoral artery occlusion. After the conventional bidirectional subintimal procedure failed, we performed two times of retrograde-DIOL fashion, and the bidirectional subintimal planes were successfully connected. After two stents implantation, a sufficient flow was achieved without complications and restenosis for two years. Case-2 involved multiple wounds in the heel due to ischemia caused by posterior tibial arterial occlusion. After the conventional bidirectional approach failed, retrograde-DIOL was performed and retrograde guidewire successfully crossed the CTO, and direct blood flow to the wounds was obtained after balloon angioplasty. The wounds heeled four months after the procedure without reintervention.

**Conclusions:**

The DIOL fashion is a useful and effective method to facilitate CTO treatment.

## Background

Endovascular re-entry techniques and devices for chronic total occlusions (CTOs) have been developed in peripheral vascular intervention (PVI). The successful intervention for peripheral artery disease (PAD) is limited by complex lesion morphology including long CTOs and severe calcification (Ingle et al., 2002). Conventional PVI failed in up to 25% femoropopliteal CTOs (Scheinert et al., 2001), and lower success rate in below-the-knee artery (BKA) lesions due to the background of patients with critical limb-threatening ischemia was reported (Tan et al., 2021).

Intimal CTO crossing cannot achieve the superiority of technical success rate and patency loss compared with subintimal crossing (Soga et al., 2013). Subintimal procedure has achieved a higher success rate of CTO crossing than intimal wiring. During CTO wiring, without the use of intravascular or extravascular ultrasound, except for calcified lesions showing the vessel path, the guidewire position is unclear. This situation may lead to unsuccessful procedures and complications such as wire perforation. To solve this problem, we propose a novel guidewire crossing with plaque modification method for PAD patients with complex occlusive lesions, named the “Direct tip Injection in Occlusive Lesions (DIOL)” fashion.

## Main text

This report is the first description of the “DIOL” fashion with hydraulic pressure through a microcatheter to expand and modify CTO lesions to facilitate guidewire crossing. This technique applies hydraulic pressure of tip injection with a contrast media through a microcatheter or an over-the-wire balloon within CTOs. The purposes of this technique are 1) to visualize the “vessel road” of the occlusion by expanding microchannels, and/or subintimal, intramedial, periadventitial space with contrast agent and 2) to modify and prepare plaques within CTO by hydraulic pressure to advance CTO devices safely and easily. The effectiveness of hydraulic pressure to crack calcifications, named the “Fracking” technique, have already been reported (Haraguchi et al., 2021). The DIOL is a procedure which can be performed from antegrade and/or retrograde approach and feature with creating dissections by hydraulic pressure. Contrast-guided subintimal tracking and re-entry (STAR), microchannel technique, and Carlino technique have already been developed as the contrast modulation techniques for difficult-to-cross CTOs in the coronary field (Azzalini et al., 2019). However, the vascular pathology and lesion characteristics in patients with coronary artery disease are different from that in PAD patients. Therefore, we should develop the appropriate approaches for PVI. Antegrade-DIOL may create dissections which extend to and compress it, especially in BKA. Hence, a gentle tip injection with smaller contrast volume (1–2 ml) should be used to confirm the tip positions which is inside or outside a vessel. On the other hand, retrograde-DIOL is used with forceful tip injection with the moderate contrast volume in up to 5-ml to visualize the vessel track and modify the plaque to facilitate CTO devices crossing. In any case, we should start a gentle injection and gradually increase the pressure while confirming the response of the lesion to the injection. We should determine the way of tip injection according to the purposes of DIOL.

**Fig. 1:**
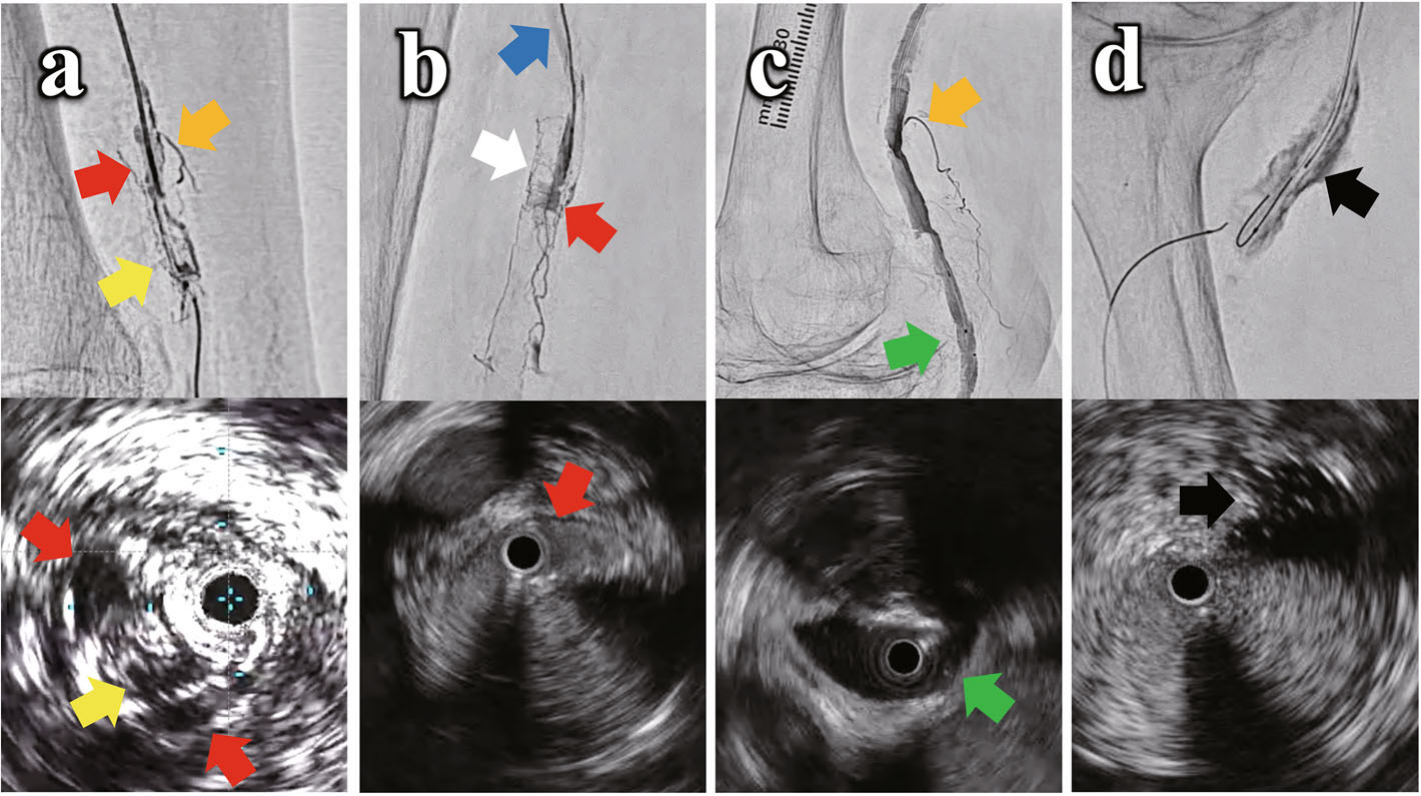
The four typical angiographic images by the “Direct tip Injection in Occlusive Lesions (DIOL)” fashion. **A** Intimal injection: microchannels which are tiny and relatively straight route without contrast stain (yellow arrow), the branch which is connected with microchannels created by an intimal injection (orange arrow), and a “tubular” dissection which is a subintimal space connected with microchannels (red arrow). **B** Subintimal injection: a “tubular” dissection (red arrow). A vasa vasorum (white arrow), a venous vasa vasorum, and veins (blue arrow), which are connected among them, are occasionally seen by a forceful injection in subintima. **C** Intramedial injection: a “river” dissection (green arrow), and the branch which is connected with intramedial space created by an intramedial injection (orange arrow). **D** Periadventitial injection: a “cloudy” contrast stain as an extravasation (black arrow)

The hydraulic pressure effect by DIOL varies among the four positions of a microcatheter tip: intima, subintima, intramedia, and periadventitia (Fig. 1). An injection in intimal plane shows microchannel routes which is tiny and relatively straight without persisting contrast stain (Fig. 1a). A “tubular” dissection results from an injection in subintimal space with a contrast stain (Fig. 1a, b). A vasa vasorum, a venous vasa vasorum, and veins which are connected among them are occasionally seen by a forceful injection in subintima (Fig. 1b). An injection in intramedial plane reveals a “river” dissection (Fig. 1c). A “cloudy” contrast stain as extravasation is caused by an injection in periadventitial plane (Fig. 1d).

**Fig. 2:**
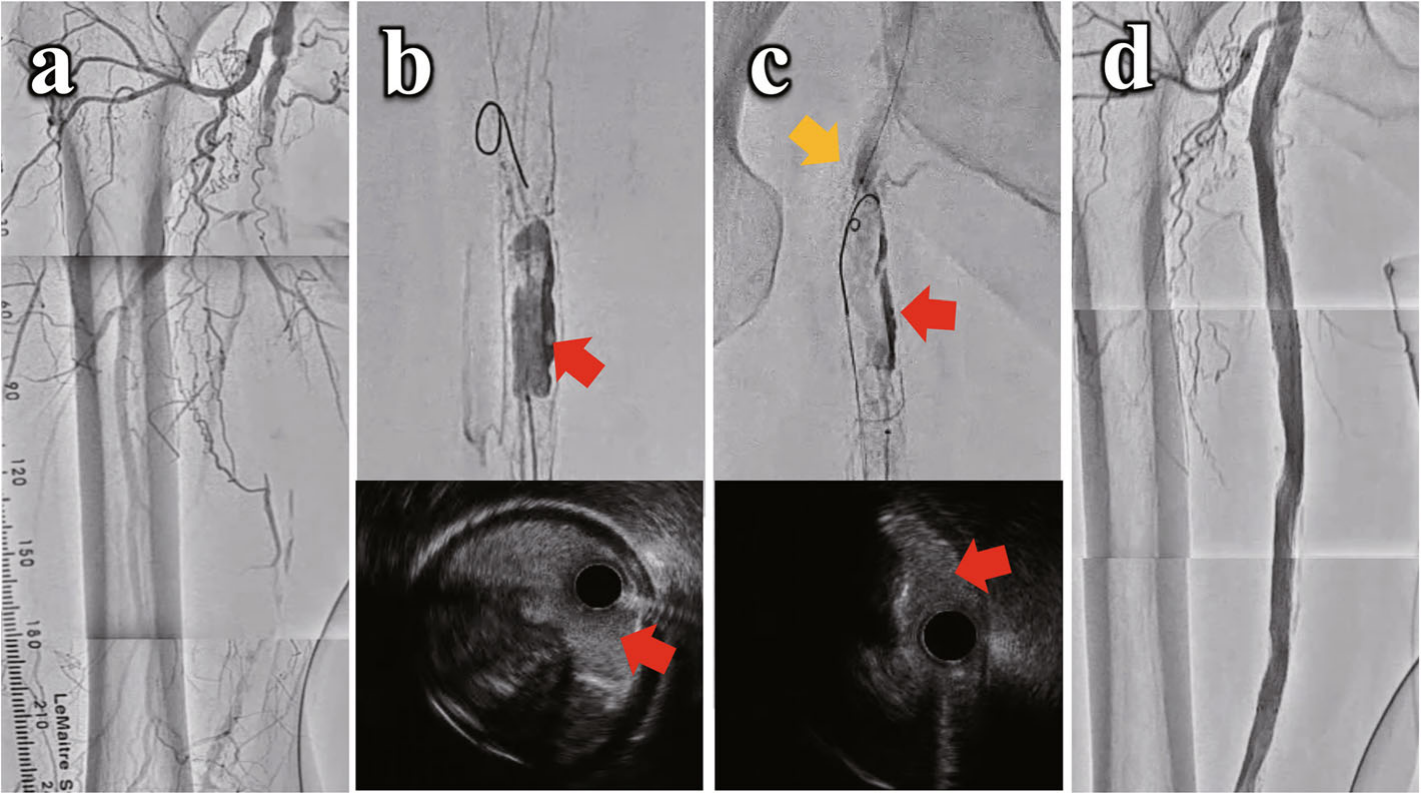
Angiography and intravascular ultrasound (IVUS) of superficial femoral arterial occlusion treatment with the “DIOL” fashion in case 1. **A** Control angiography of right superficial femoral arterial occlusion. **B** After conventional bidirectional wiring failed, retrograde-DIOL with a forceful tip injection from the retrograde microcatheter revealed a tubular dissection with a vessel road (red arrow). **C** Reverse controlled antegrade and retrograde tracking and dissection (CART) and re-entry failed. Retrograde-DIOL was reperformed to expand the subintimal lumen (red arrow). DIOL contrast penetrated into the proximal lumen and the branch (orange arrow). **D** After two drug-eluting stents deployment, a satisfactory result was achieved

Here are two cases treated by the DIOL fashion. Case-1 involved a 66-year-old male with severe claudication due to right superficial femoral artery (SFA) occlusion (Fig. 2a). Several antegrade guidewires from a 6-Fr crossover sheath only reached to the proximal CTO. A retrograde guidewire with a microcatheter inserted from distal SFA was advanced into the middle aspect of CTO, but not further. Therefore, we performed the retrograde-DIOL with a forceful tip injection from the retrograde microcatheter, and a tubular dissection with a vessel road became visible (Fig. 2b). The retrograde guidewire was successfully advanced further. We attempted reverse controlled antegrade and retrograde tracking and dissection (CART), but re-entry failed. Retrograde-DIOL was reperformed to expand the subintimal lumen and penetrate into the proximal lumen. We confirmed the connection between the lumens created by reverse-CART and retrograde-DIOL (Fig. 2c). The retrograde guidewire successfully passed the route and was advanced into the guiding sheath to achieve guidewire externalization. After two drug-eluting stents deployment, a satisfactory result was obtained without complications (Fig. 2d). Restenosis, reintervention, and amputation have not occurred two years after the treatment.

**Fig. 3:**
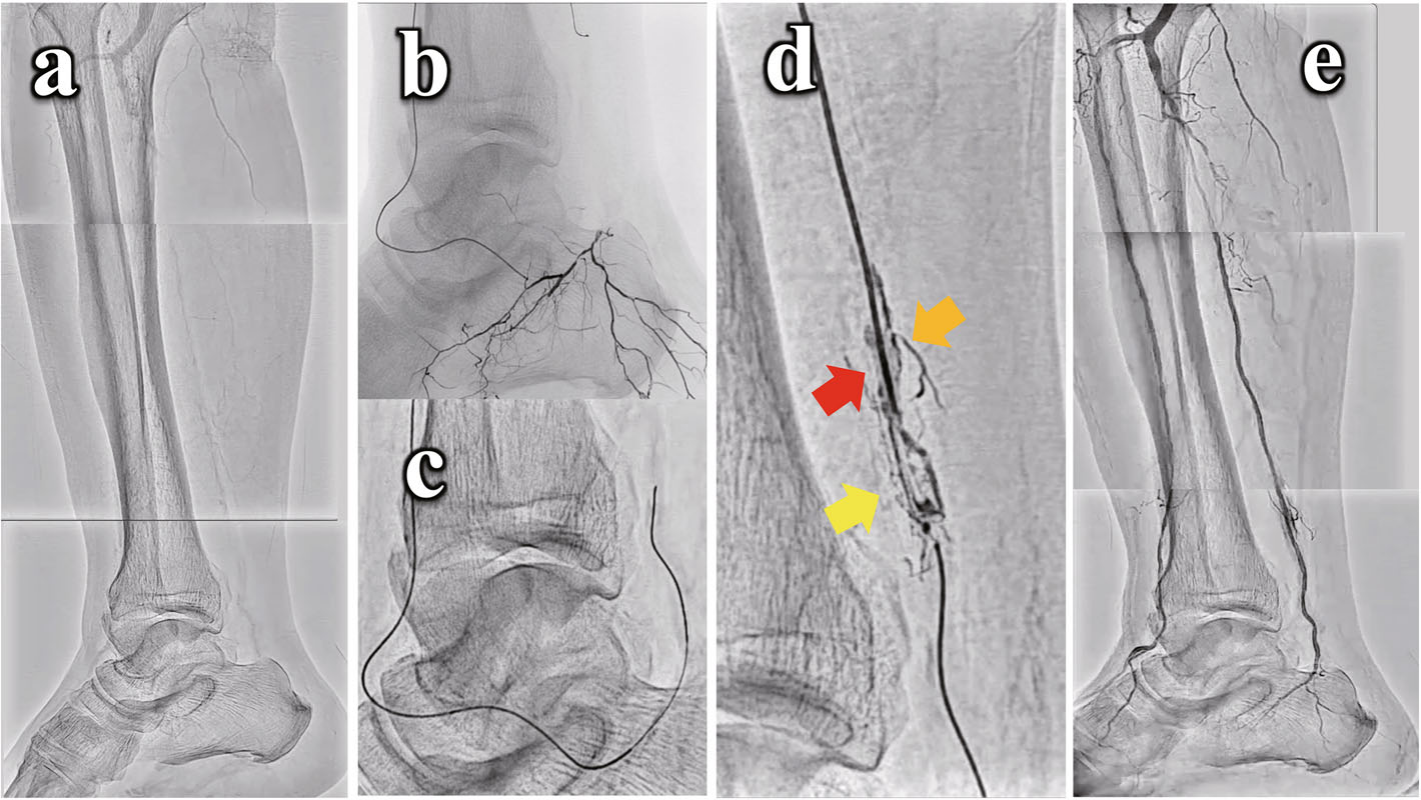
Angiography of blow-the-knee arterial occlusion treatment with the “DIOL” fashion in case 2. **A** Control angiography of below-the-knee arterial occlusions. **B**, **C**. After antegrade wiring for posterior tibial artery (PTA) occlusion, trans-collateral approach was achieved from dorsalis pedis artery through medial plantar artery to PTA. **D** Retrograde-DIOL was used to realize the position of retrograde microcatheter, visualize a vessel road, and modify the CTO plaque. Microchannels (yellow arrow), the branch (orange arrow), and a tubular dissection (red arrow) were visualized. **E** A direct blood flow to the wounds was obtained with balloon angioplasty

Case-2 involved an 83-year-old male with refractory multiple ulcers in his left heel due to BKA occlusions (Fig. 3a). Antegrade wiring for posterior tibial artery (PTA) occlusion reached distal aspect of CTO, but the crossing failed. Trans-collateral approach as retrograde approach from dorsalis pedis artery through medial lateral artery to PTA was achieved (Fig. 3b, c). The distal CTO prevented retrograde guidewire advancement. Therefore, retrograde-DIOL was used to reveal the position of retrograde microcatheter, visualize a vessel road, and modify the CTO plaque (Fig. 3d). Then, a retrograde wire successfully crossed and was advanced into the sheath to achieve guidewire externalization. After balloon angioplasty, the direct blood flow to the wounds was obtained (Fig. 3e). The wounds heeled four months after the procedure without reintervention.

Here are the limitations. First, the DIOL will not be applied if a tip of microcatheter or over-the-wire balloon following the guidewire cannot be inserted into CTO. Second, the residual contrast media around the lesion makes the angiographical image difficult to see. We should consider it as an additional treatment option when conventional procedure failed.

## Conclusions

The DIOL fashion is useful to facilitate CTO treatment. The initial results and follow-up studies with this technique compared with other re-entries are necessary to evaluate the long-term outcomes.

## Data Availability

The datasets used and/or analyzed during the current study are available from the corresponding author on reasonable request.

## Abbreviations

CTO: Chronic total occlusion
PVI: peripheral vascular intervention
PAD: Peripheral artery disease
BKA: below-the-knee artery
DIOL: Direct tip Injection in Occlusive Lesions
STAR: subintimal tracking and re-entry
SFA: superficial femoral artery
CART: controlled antegrade and retrograde tracking and dissection
PTA: posterior tibial artery
